# A FISH-Based Three-Tier Classification of Chromosome 3 Alterations in Clear Cell Renal Cell Carcinoma: Diagnostic and Prognostic Implications Utility

**DOI:** 10.3390/cancers18091460

**Published:** 2026-05-01

**Authors:** Shijie Deng, Tong Ye, Lei Zhang, Luting Zhou, Yang Liu, Yuehao Che, Lei Dong, Chaofu Wang, Xiaoqun Yang

**Affiliations:** Department of Pathology, Ruijin Hospital, Shanghai Jiao Tong University School of Medicine, Shanghai 200025, China

**Keywords:** clear cell renal cell carcinoma, 3p deletion, chromosome 3 monosomy, aneuploidy, fluorescence in situ hybridization, genomic instability

## Abstract

Background: Clear cell renal cell carcinoma (CCRCC) is the predominant subtype of renal cell carcinoma and is characterized by frequent chromosome 3 alterations, including 3p deletion, monosomy, and aneuploidy. However, the absence of standardized fluorescence in situ hybridization (FISH) cut-off values has led to inconsistent reported frequencies and limited clinical integration of this accessible assay. This study aimed to establish clinically applicable cut-off values, propose a practical three-tier classification, and evaluate its diagnostic accuracy and clinicomolecular correlation with tumor aggressiveness. Methods: FISH using VHL (3p25.3) and CEP3 probes was performed on 1748 RCC cases (1655 CCRCC, 48 papillary RCC, 45 chromophobe RCC). Cut-off values were determined by combining ROC analysis with Youden’s index and the mean + 3SD method from normal renal tubular cells. A paired cohort of 97 CCRCC cases with targeted next-generation sequencing was stratified into three subgroups (3p intact, isolated 3p loss, broad chr3 change) for clinicomolecular comparison, including 3D principal component analysis. Results: Clinically applicable thresholds of 30% for 3p deletion and 20% for monosomy identified chromosome 3 alterations in 76.9% of CCRCC cases. The combination of both markers achieved superior diagnostic accuracy (AUC = 0.82). Aneuploidy was significantly associated with higher WHO/ISUP grade (*p* < 0.001) and older age (*p* = 0.006). The three-tier classification showed stepwise progression of aggressive features (older age, higher grade, larger tumor size) and increasing PBRM1 mutation frequency from the 3p intact to the broad chr3 change group. Conclusions: This study establishes standardized FISH cut-offs and a practical three-tier classification that captures a continuous spectrum of genomic instability and tumor aggressiveness in CCRCC. Routine 3p FISH provides a simple, cost-effective, and reproducible tool with substantial diagnostic and stratification value that complements more complex genomic profiling.

## Simple Summary

Clear cell renal cell carcinoma is the most common and aggressive type of kidney cancer. Many tumors carry changes in chromosome 3 that drive cancer growth, but doctors have lacked standard ways to measure these changes using the widely available FISH test. This large study examined 1748 kidney cancer samples and established clear, practical cut-off values for detecting chromosome 3 alterations. The researchers created a simple three-tier classification system that divides tumors into three groups according to the extent of chromosome 3 changes. This system not only improves accurate diagnosis of clear cell renal cell carcinoma but also identifies which tumors are more aggressive, showing a clear progression from mild to severe disease. The findings demonstrate that routine FISH testing is a simple, inexpensive, and reliable tool that can help doctors better diagnose and stratify patients, offering practical value that complements more complex genetic tests.

## 1. Introduction

Clear cell renal cell carcinoma (CCRCC) is the most common histological subtype of renal cell carcinoma (RCC), accounting for 70–75% of all cases and representing the predominant cause of kidney cancer-related mortality worldwide [1,2,3]. Despite recent advances in targeted therapies and immunotherapy, accurate diagnosis, molecular subtyping, and risk stratification remain essential for individualized patient management. Chromosomal alterations involving chromosome 3, particularly deletion of the short arm (3p deletion) leading to biallelic inactivation of the VHL tumor suppressor gene at 3p25.3, monosomy of chromosome 3, and more complex aneuploidy, are among the most frequent and characteristic genetic events in CCRCC [1,4,5,6,7]. These alterations are believed to drive tumor initiation and progression, yet their precise frequency, optimal detection thresholds, and clinical significance continue to vary widely across studies.

Reported frequencies of 3p deletion in CCRCC range from 38% to 100%, largely attributable to differences in methodology, sample size, and the lack of standardized cut-off values [8]. Chromosome 3 monosomy and aneuploidy have been even less systematically investigated, with their occurrence and potential prognostic implications remaining largely undetermined and confined to isolated case reports [9,10]. Fluorescence in situ hybridization (FISH) has become a widely accessible and reliable technique for detecting these chromosomal abnormalities in solid tumors, including RCC. However, the absence of consensus cut-off values for key alterations such as 3p deletion and monosomy has limited its routine integration into diagnostic and prognostic workflows [8,11,12].

In this study, we performed a large-scale systematic evaluation of chromosome 3 alterations using FISH with VHL (3p25.3) and CEP3 probes in 1748 RCC cases (1655 CCRCC, 48 papillary RCC, and 45 chromophobe RCC). By combining ROC curve analysis with Youden’s index and the mean + 3SD method from normal renal tubular cells, we established practical and clinically applicable cut-off values of 30% for 3p deletion and 20% for monosomy. These thresholds enabled us to define a novel three-tier classification system—3p intact, isolated 3p loss, and broad chr3 change (encompassing monosomy or aneuploidy)—that captures a continuous biological spectrum of genomic instability. Validation in a paired cohort of 97 CCRCC cases with targeted next-generation sequencing, supported by 3D principal component analysis of clinical and mutational data, confirmed clear separation among the three subgroups and demonstrated a stepwise increase in aggressive clinicopathological features and PBRM1 mutation frequency from the 3p intact group to the broad chr3 change group.

These findings highlight the substantial diagnostic and stratification value of routine 3p FISH: a simple, cost-effective, and highly reproducible assay that reliably reflects tumor aggressiveness in a manner that is difficult to achieve through more complex genomic profiling alone. By providing standardized thresholds and a practical classification framework, this study addresses longstanding gaps in the field and supports the integration of chromosome 3 FISH into routine diagnostic and risk-assessment protocols for CCRCC.

## 2. Materials and Methods

### 2.1. Case Selection

Our study retrospectively analyzed data from patients diagnosed and treated at Shanghai Ruijin Hospital from 2016 to 2024. A total of 1748 cases of renal cell carcinoma (1655 CCRCC, 48 papillary RCC, and 45 chromophobe RCC) were included in this study. It is important to note that, at our institution, FISH testing for chromosome 3 alterations is performed as routine clinical practice for all cases diagnosed as CCRCC. In contrast, non-CCRCC subtypes (PRCC and ChRCC) are not routinely tested and were retrospectively and selectively sampled from cases diagnosed between 2023 and 2024, primarily due to cost considerations and the need to achieve adequate sample sizes for statistical comparison. This sampling strategy, while practical, may introduce selection and spectrum bias, potentially influencing the reported frequencies of chromosome 3 alterations, ROC performance, and diagnostic thresholds in non-CCRCC tumors. All diagnoses were established through consensus by at least two pathologists or confirmed by a senior genitourinary pathologist, based on comprehensive morphological evaluation, immunohistochemistry (e.g., CK7, CD10, AMACR, CAIX), and molecular methods where applicable (Supplementary Table S1).

### 2.2. Fluorescence In Situ Hybridization (FISH)

To assess chromosomal alterations on the short arm of chromosome 3 (3p), FISH was performed on formalin-fixed, paraffin-embedded tissue samples. A commercially available 3p-specific probe from Wuhan Kanglu Biotechnology Co., Ltd. (Catalog No: FP-105, Wuhan, China) was employed for the detection. This probe was designed to hybridize to the VHL gene region located at 3p25.3. Additionally, the centromere of chromosome 3 (CEP3) was marked with a green fluorophore to facilitate the identification of chromosome 3 monosomy and aneuploidy.

The FISH probe for VHL (3p25.3) and CEP3 was applied according to the manufacturer’s protocol. Hybridization signals were visualized using a fluorescence microscope (Olympus BX51, Olympus Corporation, Tokyo, Japan) equipped with an appropriate filter. VHL deletion was characterized by the deletion of the red signal relative to the green CEP3 signal, indicative of a 3p deletion. Chromosome 3 monosomy was defined by the presence of a single green signal (representing the centromere) in tumor cells.

**Figure 1:**
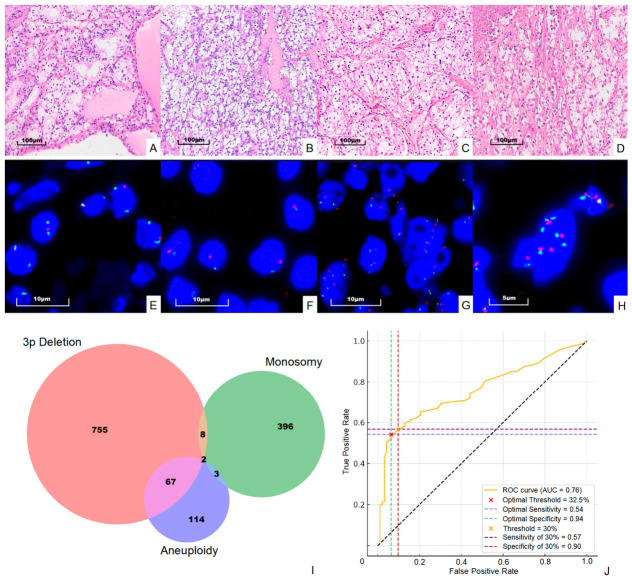
Representative FISH images and diagnostic performance of chromosome 3 alterations. (**A**–**D**) H&E staining of CCRCC with WHO/ISUP grades 1–4, respectively. (**E**–**H**) Corresponding FISH images using VHL (3p25.3, red) and CEP3 (green) probes. (**E**) Classic 3p deletion (one red, two green signals). (**F**) Chromosome 3 monosomy (one red, one green signal). (**G**,**H**) Relative 3p deletion and relative amplification, respectively. Scale bars = 100 μm (**A**–**D**), 10 μm (**E**–**G**), and 5 μm (**H**). (**I**) Venn diagram showing the distribution and overlap of 3p deletion, chromosome 3 monosomy, and aneuploidy in CCRCC cases. (**J**) ROC curve analysis demonstrating sensitivity and specificity at the Youden index-derived optimal cut-off (32.5%) and the clinically selected threshold (30%).

For each tumor sample, a minimum of 200 tumor cells were scored independently by two experienced observers to ensure reproducibility and to account for intratumoral heterogeneity. In cases demonstrating complex or heterogeneous chromosomal alterations (such as mixed 3p deletion, monosomy, and aneuploidy within the same tumor), additional cells and representative tumor regions were evaluated as appropriate to accurately capture the full spectrum of alterations. This approach is reflected in the overlapping regions observed in the Venn diagram (Figure 1I).

### 2.3. Next-Generation Sequencing (NGS) and Mutation Analysis

To investigate the molecular correlates of chromosome 3 alteration patterns, targeted next-generation sequencing (NGS) was performed on a paired cohort of 97 CCRCC cases with available FISH data. A senior genitourinary pathologist reviewed H&E-stained slides to delineate neoplastic regions ensuring ≥30% tumor cellularity. Corresponding areas were macro-dissected from 5 to 10 serial 10-μm sections of FFPE tissue blocks. Genomic DNA was extracted using the DNeasy Tissue Kit (Qiagen (Hilden, Germany)) and quantified with a Qubit Fluorometer (Thermo Fisher Scientific, Waltham, MA, USA). A minimum of 100 ng (optimal 200 ng) of DNA was used for library preparation.

Sequencing was conducted with a commercial hybrid-capture panel (Burning Rock Biotech, Guangzhou, China) covering 689 genes recurrently mutated in renal cell carcinoma and other solid tumors (including VHL, PBRM1, SETD2, BAP1, KDM5C, MTOR, and others; see Supplementary Table S6 for the full gene list). Libraries were prepared according to the manufacturer’s protocol and sequenced on an Illumina NovaSeq 6000 platform (Illumina, San Diego, CA, USA), achieving a mean depth of ~60× with >70% of target regions covered at >30×.

Raw reads were aligned to hg19 using BWA (v0.7.13), duplicate reads were marked with SAMtools (v1.3), and indel realignment was performed with GATK (v3.4). Variant calling was executed using GATK HaplotypeCaller and Mutect2, followed by annotation with ANNOVAR(version 2020-06-07). To minimize FFPE-related artifacts (particularly C > T transitions), orientation bias filtering (F1R2/F2R1) was applied. Variants were retained if they met the following criteria: mapping quality ≥30, allele frequency ≥1%, read depth ≥10, absence from population databases at AF < 0.001, and predicted pathogenicity by at least one in silico tool. Because paired normal tissue was unavailable, all non-silent variants underwent manual review by two independent investigators. Somatic mutation burden was calculated as the total number of nonsynonymous mutations per case. These data were subsequently integrated with FISH results for subclassification into three chromosome 3 alteration subgroups (3p intact, isolated 3p loss, and broad chr3 change).

### 2.4. Statistical Analysis

Statistical analyses were conducted using chi-square tests for categorical variables and Mann–Whitney U tests (or Kruskal–Wallis with Dunn’s post hoc test) for continuous variables. These analyses explored the correlations between 3p deletion, chromosome 3 monosomy, and aneuploidy with clinicopathological features such as gender, age, and WHO/ISUP grade. Additionally, the 97-case paired FISH-NGS cohort was stratified into three chromosome 3 alteration subgroups for clinicomolecular comparison. All statistical analyses were performed using R (v4.2.2) and Python (v3.8), with a two-sided *p*-value < 0.05 considered statistically significant.

To determine the cut-off values for 3p deletion and chromosome 3 monosomy, we employed ROC curve analysis with Youden’s index and the mean + 3SD method from normal renal tubule cells. Based on these approaches, we selected a balanced cut-off value of 30% for 3p deletion and 20% for monosomy. Chromosome 3 aneuploidy was defined as average CEP3 signal ≥2.5 in tumor cells.

## 3. Results

### 3.1. Cut-Off Value Calculation of FISH

To determine clinically applicable thresholds for 3p deletion, we employed two complementary statistical approaches: ROC curve analysis with Youden’s index and the mean + 3SD method from adjacent normal renal tubular cells. The ROC analysis identified an optimal cut-off of 32.5% (sensitivity 54%, specificity 94%). The mean + 3SD method yielded a lower threshold of 22%. We selected an intermediate value of 30% as a pragmatic compromise that maintains high specificity (90%) while accepting moderate sensitivity (57%) (Figure 1J).

We note that, although 3p deletion is considered a desirable diagnostic criterion in the WHO classification of CCRCC, the relatively modest sensitivity observed in our large cohort suggests that the added diagnostic utility of routine 3p FISH is limited in morphologically typical cases, where H&E-based diagnosis already achieves high accuracy. Rather than serving primarily as a diagnostic adjunct, these standardized thresholds and the resulting three-tier classification proved particularly useful for stratifying tumors according to the extent of chromosome 3 alterations and their association with aggressive clinicopathological features.

Accounting for normal biological variability, we calculated the Mean + 3SD of 3p deletion in adjacent non-cancerous renal tubule cells (see Supplementary Table S2). This calculation yielded a lower cut-off value of 22%. Alternative thresholds such as 25% were also assessed but yielded lower specificity for CCRCC diagnosis, supporting our selection of 30% for optimal balance.

Since we additionally used the centromere probe for chromosome 3 (CEP3), we were also able to assess monosomy and aneuploidy of chromosome 3. For monosomy of chromosome 3, previous studies have reported thresholds ranging from 10% to 20%. Based on considerations of normal somatic variation and the exclusion of potentially more complex chromosomal alterations (such as monosomy with amplification), we chose a more conservative cut-off value of 20%. Additionally, the criterion for identifying aneuploidy of chromosome 3 is that the average CEP3 signal in tumor cells is ≥2.5 (see Supplementary Table S2 for raw data). Figure 1 shows all four situations we evaluated in this study, including 3p deletion, monosomy of chromosome 3, 3p relative deletion, and relative amplification.

### 3.2. 3p Deletion in Renal Cell Carcinoma

FISH was performed to evaluate 3p deletion across CCRCC, PRCC, and ChRCC. A total of 1748 RCC cases were included in the analysis, comprising 1655 CCRCC cases, 48 papillary RCC, and 45 chromophobe RCC (see Supplementary Table S1). To assess the association between 3p deletion and clinicopathological features, the data were stratified by gender, age, and WHO/ISUP grade. Age was divided into two groups: ≥60 and <60 years. WHO/ISUP grading was documented on CCRCC cases. Statistical comparisons between groups were performed using the chi-square test for categorical variables and the Mann–Whitney U test, as well as the rank-sum test for continuous variables. These data were summarized in Table 1.

**Figure 2:**
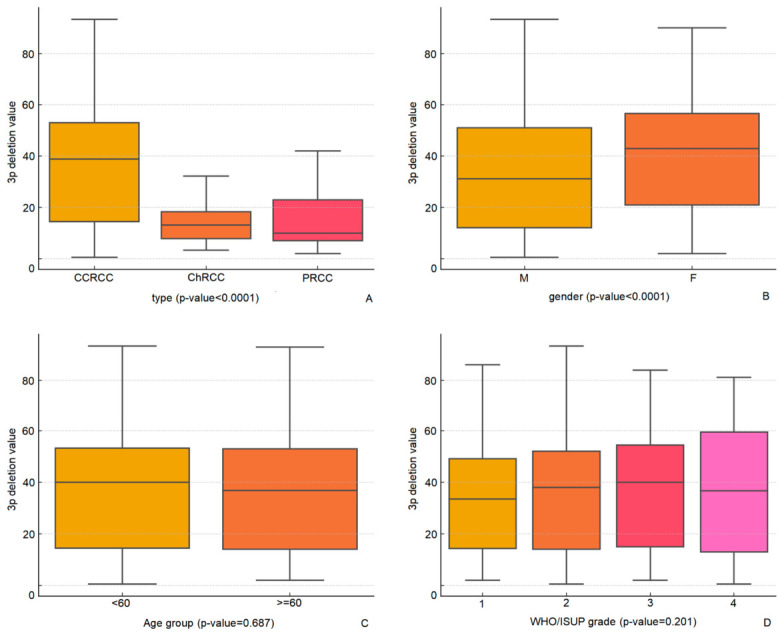
Box plots of 3p deletion values (mean signal ratios) stratified by histological subtype (CCRCC vs. PRCC vs. ChRCC), gender, age group (≥60 vs. <60 years), and WHO/ISUP grade in CCRCC cases. Statistical comparisons were performed using the Mann–Whitney U test or Kruskal–Wallis test. (**A**) Stratified by histological subtype (CCRCC, PRCC, ChRCC); (**B**) Stratified by gender (M: male, F: female); (**C**) Stratified by age group (≥60 vs. <60 years); (**D**) Stratified by WHO/ISUP grade (1–4). M = male; F = female; numbers 1–4 represent WHO/ISUP grades 1 to 4.

Among the subtypes, CCRCC had the highest frequency of 3p deletion, with 938 out of 1655 cases (56.7%) demonstrating this alteration, whereas papillary RCC and chromophobe RCC exhibited substantially lower frequencies, at 2% (1/48) and 18% (8/45), respectively. The difference in 3p deletion prevalence between CCRCC and non-CCRCC subtypes was statistically significant (*p* < 0.001). Furthermore, the 3p deletion values, indicated as mean signal ratios, were notably higher in CCRCC (35.8%) compared to chromophobe RCC (14%) and papillary RCC (18%), with a significant difference observed among the subtypes (*p* < 0.001) (Figure 2). These mean signal ratios for papillary RCC (18%) and chromophobe RCC (14%) are below the established cutoff of 30% for 3p deletion in CCRCC diagnosis (note that the Youden index-derived optimal cutoff was 32.5%, but we selected 30% as a balanced clinical threshold), which is consistent with the lower prevalence of 3p alterations in these subtypes and supports the utility of the cutoff in differentiating CCRCC from non-CCRCC tumors [13,14,15].

When stratifying by gender, 3p deletion was observed in 57.5% of male patients and 54.1% of female patients. This difference was not statistically significant (*p* = 0.222). However, the 3p deletion value was significantly higher in female patients (40.4, 95% CI: 38.5–42.3) than in male patients (33.9, 95% CI: 32.6–35.2), with a significant *p*-value of <0.001 (Figure 2).

The analysis of 3p deletion concerning age and WHO/ISUP grade did not reveal significant differences. The frequency of 3p deletion was comparable between patients aged 60 years and older (57.9%) and those younger than 60 years (55.7%), with no statistically significant difference (*p* = 0.871). The 3p deletion values between the two age groups (≥60: 35.5%; <60: 36.0%) showed no significant difference (*p* = 0.687). Similarly, when examining tumor grades, the frequency of 3p deletion did not significantly differ across the WHO/ISUP grades (*p* = 0.517). The occurrence of 3p deletion ranged from 52.9% to 58.1%. The mean 3p deletion values also demonstrated no significant variation among the different grades (*p* = 0.201), with values ranging from 33.8 to 37.1% (Figure 2).

Overall, these results indicate that 3p deletion is most prevalent in CCRCC, with a significantly higher occurrence and mean deletion value compared to other RCC subtypes. While the prevalence of 3p deletion does not significantly differ by gender, age, or tumor grade, the value of 3p deletion is notably higher in female patients.

### 3.3. Chromosome 3 Monosomy and Aneuploidy in Renal Cell Carcinoma

A total of 522 cases of CCRCC, PRCC, and ChRCC were positive for chromosome 3 monosomy (Table 2). CCRCC exhibited the highest frequency, with 515 positive cases compared to only 2 in PRCC and 5 in ChRCC (*p* < 0.001). Male patients showed a significantly higher frequency of monosomy than female patients (406 vs. 116 positive cases, *p* < 0.001), whereas no significant differences were observed according to age or WHO/ISUP grade.

**Figure 3:**
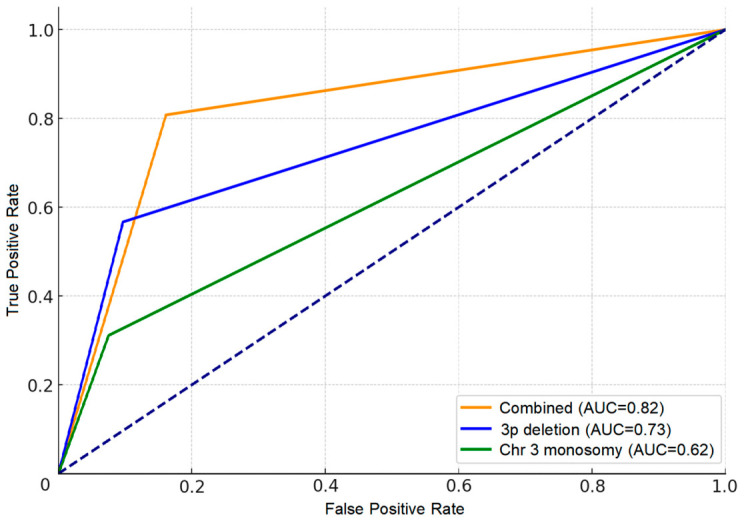
ROC curves evaluating the diagnostic performance of chromosome 3 monosomy alone (AUC = 0.62), 3p deletion alone (AUC = 0.73), and the combination of both markers (AUC = 0.82) for distinguishing CCRCC from non-CCRCC subtypes. The dotted line represents the line of no discrimination (random classifier).

To evaluate the diagnostic utility of chromosome 3 monosomy, ROC curves were generated for monosomy alone (AUC = 0.62), 3p deletion alone (AUC = 0.73), and the combination of both (AUC = 0.82). The combined use of monosomy and 3p deletion provided superior diagnostic accuracy for CCRCC compared with either marker alone (Figure 3).

**Figure 4:**
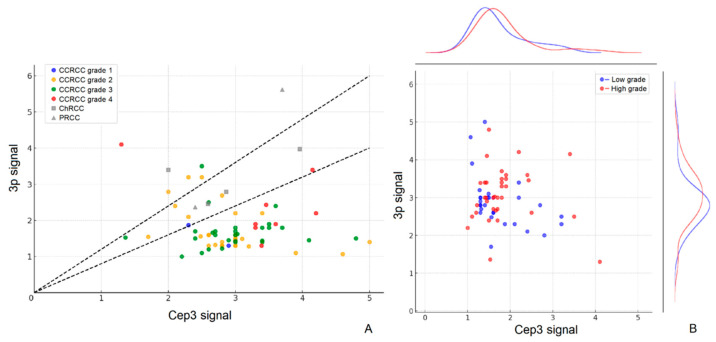
Scatter plots of 3p signal versus CEP3 signal in RCC cases. (**A**) Points are colored by histological subtype and WHO/ISUP grade. Two black dashed lines indicate the boundaries for signal ratio (0.8 and 1.2). (**B**) Points are colored by tumor grade (blue: low grade [WHO/ISUP 1–2]; red: high grade [WHO/ISUP 3–4]). Density curves on the right show the distribution of 3p signal for low-grade versus high-grade tumors.

Chromosome 3 aneuploidy was detected in 86 cases overall (Table 3 and Figure 4). CCRCC accounted for the vast majority (80 cases, 4.8%), with no significant difference in overall prevalence across subtypes (*p* = 0.537). However, aneuploidy was significantly associated with older age (6.4% in patients ≥60 years vs. 3.4% in patients <60 years, *p* = 0.006) and higher WHO/ISUP grades (8.8–8.9% in grades 3–4 vs. 1.4–2.5% in grades 1–2, *p* < 0.001). Among CCRCC cases, aneuploidy was predominantly of the relative deletion type (64 cases), whereas all 6 non-CCRCC cases showed relative amplification or borderline changes only (see Supplementary Table S3).

Collectively, monosomy and aneuploidy represent broader chromosomal alterations of chromosome 3 beyond isolated 3p deletion. While monosomy markedly improves diagnostic specificity when combined with 3p deletion, aneuploidy is strongly linked to more aggressive clinicopathological features, supporting its potential prognostic value.

### 3.4. Clinicomolecular Features of Chromosome 3 Alteration Subgroups in the Paired FISH-NGS Cohort

**Figure 5:**
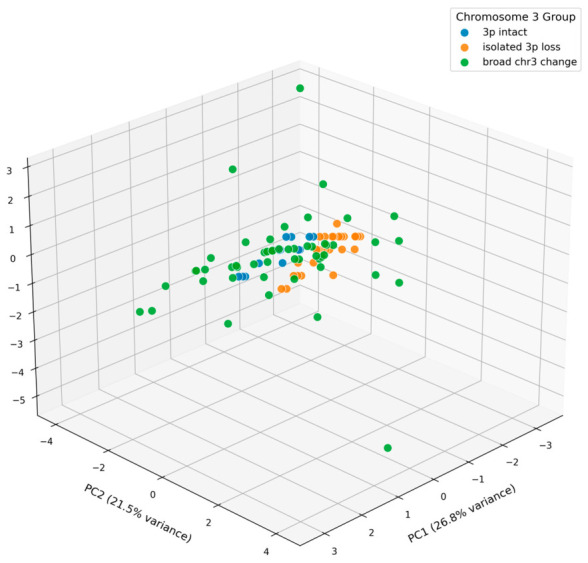
Three-dimensional principal component analysis (3D PCA) plot based on clinical parameters and somatic mutation data from the paired FISH-NGS cohort, demonstrating distinct clustering of the three chromosome 3 alteration subgroups.

**Figure 6:**
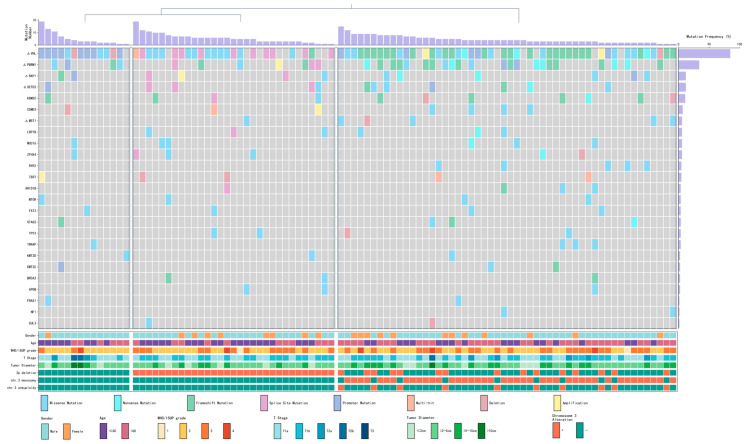
Oncoprint visualization of the top 25 most frequently mutated genes across the three chromosome 3 alteration subgroups in the 97-case cohort.

To evaluate whether the chromosome 3-based FISH classification captures clinically meaningful molecular heterogeneity, we analyzed a paired cohort of 97 CCRCC cases with both FISH and targeted NGS data. Detailed clinicopathological characteristics, sequencing panel information, and mutation results of this cohort are provided in Supplementary Table S5; mutation frequencies are listed in Supplementary Table S4; individual mutations are provided in Supplementary Table S7; the NGS gene panel is listed in Supplementary Table S6. Tumors were stratified into three subgroups: 3p intact (Group A, *n* = 14), isolated 3p loss (Group B, *n* = 31), and broad chr3 change (Group C, *n* = 52). Based on clinical case information and gene mutation data, the 3D principal component analysis (PCA) plot clearly demonstrated the separation of the three subgroups (Figure 5), confirming the robustness of this classification. The oncoprint of the top 25 mutated genes further visualized their molecular profiles (Figure 6).

As shown in Table 4, the broad chr3 change group (Group C) exhibited the most aggressive clinicopathological features, including older patient age (median 62 years, IQR 52–71; Kruskal–Wallis *p* = 0.031), the highest proportion of high-grade tumors (WHO/ISUP 3–4: 51.9% vs. 21.4% in Group A; χ^2^ *p* = 0.126), and larger tumor size (median 4.8 cm; overall *p* = 0.089). A clear stepwise increase in these aggressive features was observed from Group A to Group B to Group C.

At the molecular level, VHL mutations remained highly prevalent across all subgroups (92.9% in A, 74.2% in B, 88.5% in C), confirming their universal driver role. PBRM1 mutations (located at 3p21) showed a progressive increase toward Group C (42.3% vs. 22.6% in B and 28.6% in A; overall *p* = 0.167), consistent with broader chromosomal instability facilitating secondary hits at the 3p21 locus [16,17]. However, no other genes among the top 25 mutated genes demonstrated statistically significant differences across the three subgroups (all *p* > 0.05, see Supplementary Table S4). Notably, when comparing high-grade (WHO/ISUP 3–4) versus low-grade tumors within the entire NGS cohort, no genes showed significant differential mutation frequency, indicating that targeted sequencing alone provided limited additional information for tumor aggressiveness in this setting.

These results highlight a key advantage of FISH-based chromosome 3 assessment: while comprehensive NGS offers detailed mutational landscapes, it does not readily translate into clear stratification of tumor aggressiveness in routine practice. In contrast, the simple, cost-effective 3p FISH classification reliably captures a continuous spectrum of increasing aggressiveness from 3p intact to isolated 3p loss to broad chr3 change. This supports the clinical utility of routine 3p FISH as a practical and prognostically informative tool that complements, rather than requires replacement by, more complex genomic profiling.

## 4. Discussion

Our study provides one of the largest and most comprehensive evaluations of chromosome 3 alterations in renal cell carcinoma to date. Chromosome 3 alterations are not unique to renal cell carcinoma but represent a recurrent and clinically significant genomic event across multiple human malignancies. In uveal melanoma, monosomy 3 occurs in approximately 50% of cases and is strongly associated with metastatic disease and poor survival [18]. Recent studies further demonstrate that this deletion, often co-occurring with chromosome 8q amplification, drives extensive genomic instability and creates synthetic lethality vulnerabilities [19]. Similarly, 3p loss is one of the most frequent and earliest cytogenetic abnormalities in head and neck squamous cell carcinoma, occurring in 40–60% of cases and correlating with tumor progression [20]. Single-cell analyses have also shown that 3p deletion promotes an immunosuppressive tumor microenvironment and chemoresistance [21]. These findings highlight the broader oncogenic relevance of chromosome 3 alterations.

In renal cell carcinoma, chromosome 3 alterations coexist with other recurrent chromosomal abnormalities that vary by histologic subtype. Clear cell RCC is characterized by 3p loss in over 90% of cases, often with 5q gains. Papillary RCC frequently shows trisomy of chromosomes 7 and 17 along with Y chromosome loss. Chromophobe RCC, in contrast, exhibits multiple chromosomal losses, most commonly involving chromosomes 1, 2, 6, 10, 13, and 17 [22,23]. These subtype-specific patterns provide important context for our three-tier classification in clear cell RCC.

Through systematic FISH analysis of 1748 RCC cases, we established clinically applicable cut-off values of 30% for 3p deletion and 20% for monosomy. These thresholds identified chromosome 3 alterations in 76.9% of CCRCC cases and enabled us to define a practical three-tier classification: 3p intact, isolated 3p loss, and broad chr3 change. Validation in a paired cohort of 97 CCRCC cases with targeted NGS, supported by 3D principal component analysis, demonstrated distinct clustering of the three subgroups. This classification not only enhances diagnostic accuracy (AUC = 0.82 when combining 3p deletion and monosomy) but also captures a trend toward a continuous biological spectrum of increasing genomic instability and tumor aggressiveness, evidenced by stepwise increases in patient age, WHO/ISUP grade, tumor size, and PBRM1 mutation frequency.

Although 3p deletion is listed as a desirable diagnostic criterion in the current WHO classification, our findings indicate that the diagnostic utility of 3p FISH is more modest than previously emphasized. At the 30% threshold, sensitivity was only 57%, and even when combined with monosomy, the AUC reached 0.82. This is consistent with the fact that H&E morphology already permits accurate diagnosis in most cases. Rather than serving primarily as a diagnostic adjunct, the standardized FISH cut-offs and resulting three-tier classification provide substantial practical value for assessing tumor aggressiveness.

It should be noted that monosomy alone demonstrated limited discriminatory value (AUC = 0.62), and the non-CCRCC comparator cohort was relatively small and selectively sampled from 2023 to 2024. These factors limit the generalizability of our diagnostic metrics. Nevertheless, in real-world practice, 3p FISH is most useful in morphologically challenging cases where clear cell features are ambiguous on H&E staining, or when the differential includes clear cell papillary RCC, MiT family translocation RCC, or metastatic clear cell carcinoma. In such scenarios, the combination of 3p deletion and monosomy can provide valuable supportive evidence when integrated with immunohistochemistry and clinical context. Future prospective studies in diagnostically difficult cases are warranted to further define the optimal role of this assay.

### 4.1. Chromosome 3 Alterations Represent a Spectrum of Genomic Instability

Chromosome 3 alterations represent a spectrum of genomic instability in clear cell renal cell carcinoma. Our FISH analysis demonstrated that 3p deletion, monosomy, and aneuploidy frequently coexist, culminating in an overall alteration rate of 76.9% in CCRCC. By integrating these changes, we defined a practical three-tier classification—3p intact, isolated 3p loss, and broad chr3 change—that captures a trend toward a continuous biological spectrum from focal VHL-driven events to more extensive chromosomal instability. Although monosomy and aneuploidy (with or without concurrent 3p deletion) are not necessarily biologically equivalent, we grouped them under the “broad chr3 change” category because both subsets showed similar associations with aggressive clinicopathological features, including higher tumor grade and older age. We acknowledge that further subdivision into four or more clusters was statistically constrained by the limited size of the paired FISH-NGS cohort (*n* = 97). After exploring multiple classification schemes, we found that a three-tier system provided the optimal balance between biological interpretability and statistical robustness. This pragmatic grouping is further supported by the stepwise increase in PBRM1 mutation frequency from the 3p intact group to the broad chr3 change group in the paired NGS cohort. In the paired NGS cohort, this progression was further supported by a stepwise increase in PBRM1 mutation frequency (located at 3p21.1) from the 3p intact group (28.6%) through isolated 3p loss (22.6%) to the broad chr3 change group (42.3%), along with similar trends for other 3p21 tumor suppressors such as SETD2 and BAP1. These findings indicate that broad chromosomal alterations on chromosome 3 facilitate simultaneous inactivation of multiple key suppressors in the 3p21 region, a mechanism that FISH can readily detect but that targeted sequencing alone may not fully capture in routine practice.

### 4.2. Diagnostic and Practical Advantages of 3p FISH over Comprehensive NGS

A notable strength of this study lies in the direct comparison between simple, standardized chromosome 3 FISH assessment and more comprehensive targeted next-generation sequencing in the paired 97-case cohort. Although NGS provided a detailed mutational landscape, no robust additional associations were detected between mutation frequencies and tumor grade or chromosome 3 alteration subgroups in this dataset (except for a non-significant increasing trend in PBRM1). This indicates that extensive genomic profiling, while informative for research purposes, does not readily translate into clear, clinically actionable stratification of tumor aggressiveness in routine practice. In contrast, the rigorously determined cut-off values for 3p deletion (30%) and monosomy (20%)—derived from ROC analysis combined with mean + 3SD from normal renal tubular cells—enabled FISH to effectively capture a continuous biological spectrum of increasing tumor aggressiveness, from 3p intact to isolated 3p loss to broad chr3 change [24,25,26,27]. When 3p deletion and monosomy were combined, diagnostic accuracy for CCRCC reached an AUC of 0.82, substantially higher than either marker alone. These findings underscore a key practical advantage: a simple, cost-effective, and highly reproducible FISH assay can reliably reveal the progressive enhancement of tumor aggressiveness, offering significant clinical utility that is difficult to achieve through more complex sequencing approaches alone.

### 4.3. Prognostic Implications and Clinical Translation

The three-tier classification captured a trend toward increasing aggressive clinicopathological features (older age, higher WHO/ISUP grade, larger tumor size) and higher PBRM1 mutation frequency from the 3p intact group to the broad chr3 change group. These associations align with our findings in the main 1748-case FISH cohort, where chromosome 3 aneuploidy was strongly linked to higher tumor grades (*p* < 0.001) and older age (*p* = 0.006) [9,10,16,28]. Collectively, these data suggest that broad chromosomal alterations on chromosome 3 reflect a more advanced state of genomic instability and may serve as a useful marker for risk stratification in CCRCC.

From a clinical translation perspective, the FISH-based three-tier classification offers a simple, reproducible, and cost-effective framework for risk stratification in CCRCC, analogous to the HER2/CEP17 ratio system widely used in breast cancer [29,30]. Although long-term follow-up data were not available in the current study, the consistent correlation between broad chr3 change and aggressive clinicopathological parameters supports the potential of routine 3p FISH to refine risk assessment and complement morphological evaluation in daily practice.

### 4.4. Limitations and Future Directions

Despite the strengths of this large-scale analysis, several limitations should be acknowledged. In particular, the retrospective single-center design and the institution-specific testing policy (routine 3p FISH only for CCRCC, with non-CCRCC cases selectively sampled from 2023 to 2024 for cost-related reasons) fundamentally shaped the cohort composition. This approach, while practical, introduces a risk of selection and spectrum bias and limits the generalizability of our diagnostic performance metrics to broader populations. The selective sampling of non-CCRCC cases may have influenced the reported frequencies of chromosome 3 alterations and the ROC performance in the comparator group. First, although the FISH cohort comprised 1748 cases, the paired NGS validation was limited to 97 cases from a single institution, which may restrict generalizability. Second, long-term follow-up data were not available, preventing direct assessment of the prognostic impact of the three-tier classification on progression-free or overall survival. Third, the targeted NGS panel, while comprehensive for known RCC driver genes, did not cover the entire genome, potentially missing broader structural variants or epigenetic alterations. Finally, functional validation of how broad chr3 changes contribute to aggressive behavior remains to be explored.

Future studies should address these gaps through multi-center validation cohorts with extended clinical follow-up. Integration of whole-genome sequencing and functional experiments (e.g., CRISPR-based modeling of simultaneous 3p21 gene inactivation) will further elucidate the mechanistic basis of the observed spectrum. Prospective trials evaluating whether routine 3p FISH can guide risk-adapted surveillance or therapy intensification are also warranted.

## 5. Conclusions

Our study demonstrates that chromosome 3 alterations, readily detected by standardized FISH with clinically validated cut-offs, capture a biologically and clinically meaningful continuum of genomic instability and tumor aggressiveness in CCRCC. The simple, cost-effective nature of 3p FISH provides substantial stratification value that complements more complex genomic profiling, rather than being replaced by it. We propose that incorporation of this three-tier FISH classification into routine diagnostic workflows and future classification systems will improve precision in CCRCC risk assessment and clinical management.

## Figures and Tables

**Table 1:** 3p deletion frequency across RCC subtypes, gender, age, and WHO/ISUP grades.

		3p Deletion num.		*p* Value	3p Deletion Value% (95% CI)	*p* Value
		Positive (%)	Negative (%)			
subtype				<0.001		<0.001
PRCC		1 (2.1%)	47 (97.9%)		17.78 (12.52–23.03)	
ChRCC		8 (17.8%)	37 (82.2%)		13.56 (11.39–15.72)	
CCRCC		938 (56.7%)	717 (43.3%)		35.75 (34.66–36.85)	
	Gender			0.222		<0.001
	Male	709 (57.5%)	523 (42.5%)		33.92 (32.61–35.24)	
	Female	229 (54.1%)	194 (45.9%)		40.42 (38.52–42.33)	
	Age			0.871		0.687
	≥60	425 (57.9%)	309 (42.1%)		35.52 (33.93–37.11)	
	<60	513 (55.7%)	408 (44.3%)		35.97 (34.46–37.48)	
	WHO/ISUP grade			0.517		0.201
	Grade 1	74 (52.9%)	66 (47.1%)		33.75 (30.17–37.33)	
	Grade 2	599 (56.6%)	460 (43.4%)		35.23 (33.87–36.59)	
	Grade 3	198 (58.1%)	143 (41.9%)		37.05 (34.61–39.48)	
	Grade 4	48 (53.3%)	42 (46.7%)		36.48 (31.53–41.43)	

**Table 2:** Chromosome 3 monosomy frequency.

		Chromosome 3 Monosomy		*p* Value
		Positive (%)	Negative (%)	
subtype				<0.001
PRCC		2 (4.4%)	43 (95.6%)	
ChRCC		5 (10.4%)	43 (89.6%)	
CCRCC		515 (31.1%)	1140 (68.9%)	
	Gender			<0.001
	Male	406 (32.5%)	843 (67.5%)	
	Female	116 (23.2%)	383 (76.8%)	
	Age			0.399
	≥60	260 (30.9%)	582 (69.1%)	
	<60	262 (28.9%)	644 (71.1%)	
	WHO/ISUP grade			0.093
	Grade 1	44 (30.8%)	99 (69.2%)	
	Grade 2	344 (31.9%)	733 (68.1%)	
	Grade 3	108 (30.3%)	248 (69.7%)	
	Grade 4	18 (19.4%)	75 (80.6%)	

**Table 3:** Chromosome 3 aneuploidy frequency.

		Chromosome 3 Aneuploidy		*p* Value
		Positive (%)	Negative (%)	
subtype				0.537
PRCC		2 (4.4%)	43 (95.6%)	
ChRCC		4 (8.3%)	44 (91.7%)	
CCRCC		80 (4.8%)	1575 (95.2%)	
	Gender			0.804
	Male	56 (4.7%)	1133 (95.3%)	
	Female	24 (5.2%)	442 (94.8%)	
	Age			0.006
	≥60	51 (6.4%)	745 (93.6%)	
	<60	29 (3.4%)	830 (96.6%)	
	WHO/ISUP grade			<0.001
	Grade 1	2 (1.4%)	138 (98.6%)	
	Grade 2	27 (2.5%)	1032 (97.5%)	
	Grade 3	30 (8.8%)	311 (91.2%)	
	Grade 4	8 (8.9%)	82 (91.1%)	

**Table 4:** Clinicopathological characteristics according to chromosome 3 alteration subgroups in the paired FISH-NGS cohort. Bold values indicate statistically significant differences (*p* < 0.05).

	Group (*n* = 97)			*p*-Value			
Characteristic	Group A (*n* = 14)	Group B (*n* = 31)	Group C (*n* = 52)	Overall	A vs. B	A vs. C	B vs. C
Age (IQR)	53 (43–65)	59 (48–68)	62 (52–71)	**0.031**	0.142	**0.012**	0.218
Age > 60, *n* (%)	5 (35.7)	15 (48.4)	31 (59.6)	0.168	0.412	0.085	0.312
Male, *n* (%)	10 (71.4)	23 (74.2)	41 (78.8)	0.742	0.845	0.512	0.623
WHO/ISUP grade3/4, *n* (%)	3 (21.4)	14 (45.2)	27 (51.9)	0.126	0.118	0.068	0.521
Tumor size (IQR)	3.5 (2.5–4.8)	4.0 (3.0–5.5)	4.8 (3.8–6.2)	0.089	0.231	**0.041**	0.187
Mutation count (IQR)	3 (2–5)	4 (2–6)	5 (3–7)	0.214	0.392	0.098	0.245
T stage, T1a+b, *n* (%)	11 (78.6)	24 (77.4)	37 (71.2)	0.712	0.923	0.512	0.489

## Data Availability

The data presented in this study are available on reasonable request from the corresponding authors.

## Supplementary Materials

The following supporting information can be downloaded at: https://www.mdpi.com/article/10.3390/cancers18091460/s1, Supplementary Table S1: Clinicopathological and FISH characteristics of the entire 1748 RCC cohort; Supplementary Table S2: 3p deletion ratios in normal renal tubular epithelial cells used for cut-off calculation (Mean + 3SD method); Supplementary Table S3: Detailed patterns of chromosome 3 aneuploidy (deletion, amplification, borderline) across subtypes, gender, age, and WHO/ISUP grade; Supplementary Table S4: Mutation frequencies of the top 25 genes across the three chromosome 3 alteration subgroups in the paired FISH-NGS cohort (*n* = 97); Supplementary Table S5: Detailed clinicopathological and FISH data of the 97 paired FISH-NGS CCRCC cases; Supplementary Table S6: Complete gene list of the 689-gene targeted next-generation sequencing panel; Supplementary Table S7: Individual somatic mutations detected in the 97 paired FISH-NGS cases.
